# Performance of case-control rare copy number variation annotation in classification of autism

**DOI:** 10.1186/1755-8794-8-S1-S7

**Published:** 2015-01-15

**Authors:** Worrawat Engchuan, Kiret Dhindsa, Anath C Lionel, Stephen W Scherer, Jonathan H Chan, Daniele Merico

**Affiliations:** 1 Data and Knowledge Engineering Laboratory, School of Information Technology, King Mongkut's University of Technology Thonburi, Bangkok 10140, Thailand; 2 Neurotechnology and Plasticity Lab, School of Computational Science and Engineering, McMaster University, Hamilton, Ontario L8S 4L8, Canada; 3 Program in Genetics and Genome Biology, The Centre for Applied Genomics, The Hospital for Sick Children, Toronto, Ontario M5G 0A4, Canada; 4 Department of Molecular Genetics, University of Toronto, Toronto, Ontario M5S 1A8, Canada; 5 McLaughlin Centre, University of Toronto, Toronto, Ontario M5G 0A4, Canada; 6 Centre of Excellence in Genomic Medicine Research (CEGMR), King Abdulaziz University, P.O. Box: 80216, Jeddah 21589, KSA

**Keywords:** Copy number variation (CNV), Autism Spectrum Disorders (ASD), rare genetic variants, machine learning classification, Random Forest (RF)

## Abstract

**Background:**

A substantial proportion of Autism Spectrum Disorder (ASD) risk resides in *de novo* germline and rare inherited genetic variation. In particular, rare copy number variation (CNV) contributes to ASD risk in up to 10% of ASD subjects. Despite the striking degree of genetic heterogeneity, case-control studies have detected specific burden of rare disruptive CNV for neuronal and neurodevelopmental pathways. Here, we used machine learning methods to classify ASD subjects and controls, based on rare CNV data and comprehensive gene annotations. We investigated performance of different methods and estimated the percentage of ASD subjects that could be reliably classified based on presumed etiologic CNV they carry.

**Results:**

We analyzed 1,892 Caucasian ASD subjects and 2,342 matched controls. Rare CNVs (frequency 1% or less) were detected using Illumina 1M and 1M-Duo BeadChips. Conditional Inference Forest (CF) typically performed as well as or better than other classification methods. We found a maximum AUC (area under the ROC curve) of 0.533 when considering all ASD subjects with rare genic CNVs, corresponding to 7.9% correctly classified ASD subjects and less than 3% incorrectly classified controls; performance was significantly higher when considering only subjects harboring *de novo* or pathogenic CNVs. We also found rare losses to be more predictive than gains and that curated neurally-relevant annotations (brain expression, synaptic components and neurodevelopmental phenotypes) outperform Gene Ontology and pathway-based annotations.

**Conclusions:**

CF is an optimal classification approach for case-control rare CNV data and it can be used to prioritize subjects with variants potentially contributing to ASD risk not yet recognized. The neurally-relevant annotations used in this study could be successfully applied to rare CNV case-control data-sets for other neuropsychiatric disorders.

## Background

Autism Spectrum Disorders (ASD) affect about 1% of the population, with a higher prevalence in males than females, and are characterized by impairments in social interaction and communication, as well as by repetitive and restricted behavior [1,2]. ASDs are highly heritable [3] and genomic studies have revealed that a substantial proportion of ASD risk resides in *de novo* germline and rare inherited genetic variation, ranging from chromosome abnormalities and copy number variation (CNV) [4-8] to single nucleotide variation [9-14]. Genomic studies have highlighted a striking degree of genetic heterogeneity, with variation distributed across numerous genes enriched in synaptic components as well as broader neuronal processes and neurodevelopmental pathways [15]. While numerous ASD loci have been recognized to date, they only account for a small fraction of the overall estimated heritability [16], consistent with the prediction that there are about 1000 loci underlying ASD [17].

Rare CNVs are expected to contribute to ASD risk in up to 10% of ASD subjects [15], whereas estimates for rare nucleotide substitutions and small insertions/deletions range between 10 and 40% for different studies (depending on the sequencing technology, the modes of inheritance investigated and the depth of phenotypic characterization) [9-14].

Certain multigenic regions with rare yet recurrent inherited or *de novo* CNVs (genomic disorder loci such as 15q11.2-q13 duplications and 16p11.2 deletion/duplication), as well as rare variants disrupting single genes (such as *NRXN1*), have a well established contribution to ASD etiology. *De novo* CNVs are observed in up to 5-10% of screened ASD subjects; however, not all of these events have a clear contribution to ASD risk, which is thought to depend on the size of the genomic change and the gene pathways perturbed. In this sample collection, 3.0% of ASD subjects harbored a *de novo* or inherited genic CNVs classified as pathogenic according to clinical annotation guidelines [18] and consensus catalogue of ASD loci (124 genes and 55 loci) [16]; more than half of these pathogenic CNVs were *de novo*.

While univariate statistics are well suited for investigating global burden and for discovering pathways implicated in ASD, multivariate machine learning methods are best suited to achieve optimal classification performance using all available gene annotations. Data-driven classification is complementary to CNV clinical classification based on established ASD genetics, and thus helps expanding the number of “explained” subjects. Unlike other published studies focusing uniquely on pathogenic CNV classification [19], we aimed at classifying subjects based on the contribution of all rare CNVs.

Many classification algorithms have been proposed to solve different classification problems. Random Forest (RF) is an ensemble learning method that can be used for binary classification as well as for regression analysis. A RF classifier is composed of a large number of decision trees, each trained using a random subset of samples (bagging) and constructed by selecting the best splitting features from random and independent sampled subsets of features [20,21]. RF has been used in different fields, including computational biology; it is robust, accurate, with limited or no overfitting issues, and with transparent feature utilization. Despite its robustness, the relevance measures used by RF to select the best splitting features have been shown to be positively biased towards features with a larger number of categorical values; additional shortcomings occur in presence of highly correlated variables [22]. To avoid these issues, the Conditional Inference Forest (CF) classification method was proposed as a modification of RF that utilizes statistical inference testing for feature selection during tree construction [23].

Support Vector Machines (SVM) [24] and Artificial Neural Networks (NN) [25] are two popular classification methods, based on different mathematical models than Random Forest. SVM uses its kernel to project the data points from the feature space to a higher dimensional space, where a hyperplane is sought to maximize the separation between the nearest data points from each class (also called the support vectors). Artificial Neural Networks, in particular Multilayer Perceptrons (here called simply Neural Networks, or NNs) are built up of three or more layers of nodes and weighted connections between them; weights are set maximizing the rate at which the output layer correctly identifies the true class of given examples.

In this work, we investigated classification performance of different methods and the corresponding fraction of ASD subjects who can be reliably classified as cases.

## Methods

### ASD sample collection

Samples were collected as part of the Autism Genome Project (AGP), an international consortium with more than fifty contributing sites in North America and Europe; informed consent was obtained from all participants. ASD was diagnosed based on the ADOS (Autism Diagnostic Observation Schedule) and ADI-R (Autism Diagnostic Interview - Revised) [26,27]. Patients with karyotypic abnormalities, Fragile X syndrome or other genetic syndromes causing congenital malformations were excluded. Only samples from subjects with European ancestry were used for this analysis, as determined by multidimensional scaling analysis (MDS) of known SNPs (single nucleotide polymorphisms).

Unrelated controls were assembled from three large studies in which subjects had no previous psychiatric history: SAGE (Study of Addiction Genetics and Environment), Ontario Colorectal Cancer study, HABC (Health Aging and Body Composition) [28-31]. Controls were genotyped on the same array platforms (Illumina 1M single or duo arrays) as ASD subjects and parents, applying the same quality control procedures and CNV calling algorithms.

Part of the samples (stage 1) was analyzed and published in 2010 [8], whereas the remainder (stage 2) is being published in a separate paper [32].

### CNV calling and clinical classification

Only samples meeting quality thresholds were used for CNV analysis. CNVs (of size 30 kb or greater) were detected using an analytical pipeline optimized for Illumina 1M arrays [8,33]. All *de novo* CNVs were experimentally validated. Samples with copy number variation greater than 7.5 MB were excluded.

The clinical classification of *de novo* and inherited CNVs as pathogenic, uncertain or benign was established according to the American College of Medical Genetics guidelines [18], on the basis of genetic loci known to be implicated in ASD (124 genes and 55 loci) [16]; large and very rare CNVs were also classified as pathogenic, even if they were not reported before. Experimental CNV validation details can be found in [33].

### CNV gene annotations and gene-set construction

Rare CNVs were mapped to genes whenever at least one transcript overlapped the CNV; transcript coordinates were based on RefSeq hg18 (the same build as for the BeadChip arrays). Similar performance results were obtained using the more stringent exonic mapping (i.e. requiring at least one exon to be overlapped by a CNV).

Curated neurally-relevant gene-sets captured (i) predicted haploinsufficiency [33], (ii) brain expression levels, based on the spatiotemporal Brainspan RNA-seq data-set [35], as well as brain expression specificity compared to other tissues, based on the Novartis Tissue Atlas microarrays [36], (iii) experimentally-determined synaptic complex membership [37] and regulation by *FMR1*, a key modulator of mRNA translation required for synaptic plasticity, based on two different methods [38,39], (iv) implication in neurological or neuropsychiatric disease in humans (according to HPO, the Human Phenotype Ontology) [40], (v) implication in abnormal nervous system or abnormal behavior in mice, according to Mammalian Phenotype Ontology (MPO) annotations provided by Mouse Genome Informatics (MGI) [41], and (vi) neuronal or nervous system function based on Gene Ontology (GO) [42] annotations or pathway membership. A more detailed description is provided in the supplementary methods and results [see Additional file 1].

### Cross-validation strategy

Stratified three-fold cross-validation was used to avoid overfitting [43]. Subjects with at least one rare CNV were randomly divided into three equal subsets, each with the same proportion of cases and controls. The union of two subsets was used to train the model, while the remaining subset was used as the test set, to assess the classification performance. This process was repeated three times without re-dividing the data-set, so that each subset was used once as test set and twice as training set. Twenty independent cross-validation iterations were performed to estimate the mean and standard deviation of the area under the curve (AUC), to model the classification stochasticity. Absence of overfitting was further assessed by replacing real classification features with randomized features based on gene identity permutation.

### Classifier implementation details

For RF, we used the implementation provided by the R/CRAN package ‘randomForest’ version 4.6-7 [44], which follows the original algorithm proposed by Breiman 2001 [20]. We used default settings unless otherwise specified. For CF, we used the implementation provided by the R/CRAN package ‘party’ version 1.0-9 [45]. We used default settings unless otherwise specified. R 2.15.2 was used for all RF and CF analyses.

For the linear SVM, the libSVM package was used [46] in MATLAB R2013a. The cost parameter was kept at default as 1 and class weights were kept even. Each feature was independently normalized and rescaled to a 0-1 interval prior to being input into the classifier.

The Neural Network was built with two middle layers of 100 and 50 nodes each, a learning rate of 0.005 (which affects the rate of connection weight adaptation) with a 0.9 momentum (which affects the acceleration of connection weight adaptation). The network was trained through back-propagation, and unlike the SVM, it did not require feature normalization or scaling. The NN was also implemented in MATLAB R2013a.

### Feature relevance metrics and feature selection

Two feature relevance metrics were utilized for RF: Mean Decrease Accuracy (MDA) and Mean Decrease Gini, as implemented in the randomForest package. MDA is calculated by permuting the value of the feature in OOB (out of bag) samples and comparing the accuracy of prediction before and after permutation tree by tree. Mean Decrease Gini is a measure of how each feature contributes to the homogeneity of the nodes and leaves.

MDA was also used for CF, as implemented by the party package function ‘varimp’, using the standard version (unless stated otherwise); in fact, the conditional version, which adjusts for correlations between predictor variables, is extremely computationally demanding.

For MDA-based selection, we ranked features based on MDA, and selected a predefined number of features. The procedure used for step-wise decorrelation and the detailed implementation of Minimum Redundancy Maximum Relevance Feature Selection (MRMR) [46] are described in detail in the supplementary methods and results [see Additional file 1].

### Percentage of correctly classified ASD subjects

The percentage of correctly classified ASD subjects was calculated as the number of ASD subjects correctly predicted in at least 15 out of 20 iterations divided by the study total (1,892); the prediction probability cutoff was chosen ensuring that the percentage of correctly predicted ASD subjects without pathogenic or *de novo* CNVs exceeds the percentage of incorrectly predicted controls by more than 1.5x. Carriers of pathogenic and *de novo* CNV are easier to classify, thus the ratio described above is a more conservative estimate of the true positive to false positive ratio. Results did not change substantially when requiring subjects to be classified in at least 10 out of 20 iterations.

## Results and discussion

### Feature construction

We analyzed 1,892 ASD subjects (1623 males and 270 females) and 2,342 platform-matched controls (1093 males and 1250 females) with at least one rare CNV (frequency 1% or less); all subjects are of Caucasian ethnicity. Rare CNVs were mapped to gene transcripts; *de novo* and inherited rare CNVs were labeled as pathogenic, uncertain or benign following clinical annotation guidelines. Univariate burden and pathway analysis, as well as details on the clinical CNV classification, can be found in a separate publication [32].

Classification features were constructed for every subject as gene counts for each gene-set, i.e. counting how many genes participating in a gene-set harbor a rare genic gain or loss; in particular, separate features were constructed for gains and losses. For CNV mapping, we decided to focus on transcripts of known genes as there is a wealth of information that can be used to predict their implication in autism, and their boundaries are well characterized in the human genome; upstream transcription start site regulatory motifs, or other non-coding sequence could be the object of another paper.

Only subjects harboring at least one rare genic CNV were used for classification, as features would be constantly zero for the other subjects, but all subjects were considered when reporting percentage “explained” statistics. This resulted in a subset of 1,570 ASD subjects (80.8%) and 1,916 controls (81.8%), of which 958 ASD subjects and 1,113 controls had at least one genic loss, whereas 1,132 ASD subjects and 1,363 controls had at least one genic gain. 78 ASD subjects harbored at least one genic *de novo* CNV (30 gains, 51 losses); 57 ASD subjects subjects harbored at least one *de novo* or inherited CNV recognized as “pathogenic” according to clinical significance annotation [16,18] (21 gains, 37 losses). All subsets presented a gender composition similar to the full data-sets.

Gene-sets (and corresponding classification features) were organized in three groups: (a) 20 curated gene-sets of neurobiological relevance, capturing brain expression, synaptic components, neuro-phenotypes in human and mouse and predicted haploinsufficiency (Table 1), (b) gene-sets corresponding to GO annotations, (c) gene-sets corresponding to pathways (KEGG, Reactome, NCI Pathway Interaction Database, Biocarta databases) [48-53]. GO and pathway gene-sets were filtered to remove exceedingly large or small sets, resulting in 1425 GO sets (out of 5657) with 100 to 3000 genes, and 519 pathway sets (out of 1763) with 50 or more genes. The total gene count, regardless of gene-set membership, was also used as a classification feature.

**Table 1:** Curated gene-sets description and gene number

Gene-set ID	Gene-set Description	Gene N#
hi015	Predicted haploinsufficiency (most inclusive)	8862

hi035	Predicted haploinsufficiency	4136

hi055	Predicted haploinsufficiency (most stringent)	2214

ExpsNov_BrainFeAd_sp	Specific expression in human adult or fetal brain (Novartis Tissue Atlas)	1285

Synapse_GrantFull	Post-synaptic density components	1407

FMR1_Targets_Darnell	FMR1 targets (Darnell et al)	840

FMR1_Targets_Ascano	FMR1 targets (Ascano et al)	927

thrEXPR_log2rpkm	Expressed in brain (BrainSpan)	13802

thr4.86_log2rpkm	Expressed in brain, very high (BrainSpan)	4595

thr3.32_log2rpkm	Expressed in brain, high/medium (BrainSpan)	4604

thr0.84_log2rpkm	Expressed in brain, medium/low (BrainSpan)	4603

thr.MIN_log2rpkm	Not expressed in brain (BrainSpan)	4600

PhHs_NervSys_ADX	Human nervous system phenotype (HPO), autosomal dominant or X-linked	620

PhHs_NervSys_All	Human nervous system phenotype (HPO)	784

PhHs_MindFun_ADX	Higher mental function phenotype (HPO), autosomal dominant or X-linked	395

PhHs_MindFun_All	Higher mental function phenotype (HPO)	687

MmHs_Neuro_All	Mouse neuro phenotype (MGI/MPO)	3479

MmHs_Extend_All	Mouse developmental phenotype (MGI/MPO)	4314

NeuroF_large	Neurobiological function, inclusive	2601

NeuroF_small	Neurobiological function, stringent	1088

Total	Total gene count	18203

For different classifiers and parameter settings, we classified all ASD subjects, or only the ASD subjects carrying a pathogenic or *de novo* rare CNV. This was helpful to evaluate the performance of our classification approach for more obviously implicated genes and loci. In addition, we classified either all ASD subjects with any rare genic CNV, or only the carriers of rare genic losses (using only loss-based features) or rare genic gains (using only gain-based features). This was helpful to evaluate the predictive power of the two CNV types.

### Classification results: RF and CF

**Figure 1:**
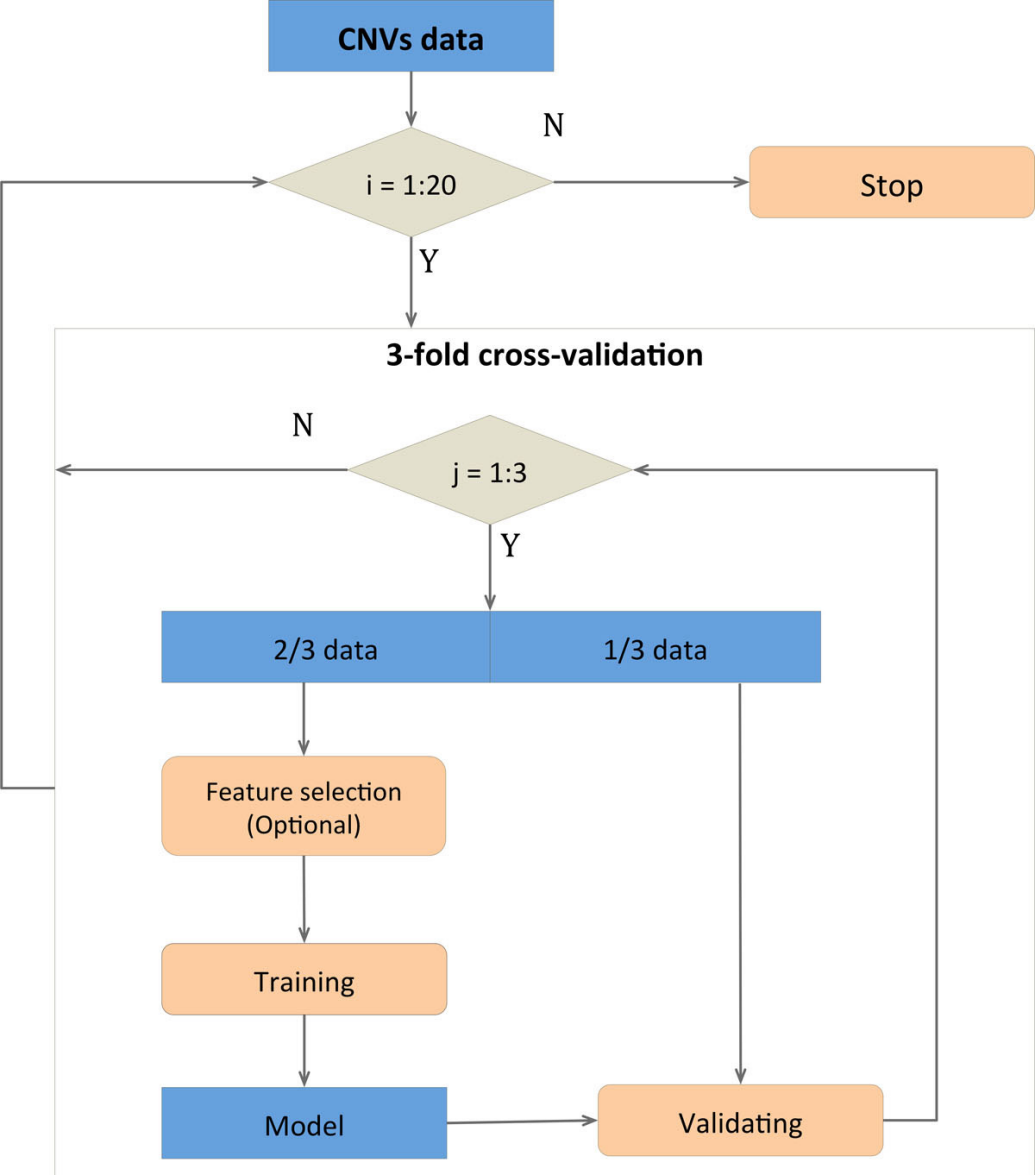
**Cross-validation strategy.** The data-set is divided into three equal subsets, each with the same propotion of ASD and control subjects. Two of the tree subsets are used as the training set the model, whereas the other subset is used as the validation set for performance quantification; this is iterated three times, so that each subset is used twice for training and once for validation. The feature selection is performed only for GO and pathway-based features. The remaining set is used as test set to assess the performance of classification. The cross-validation procedure is repeated times to estimate the mean performance and its standard deviation.

For all classifiers, we used a robust cross-validation approach and tested randomized features to ensure the absence of significant overfitting (Figure 1).

Random Forest (RF) was our original choice for its resilience to overfitting and its capability to handle a large feature space [20]. However RF has been criticized for its positive selection bias towards features with more categorical values [22]; for this reason, Conditional Inference Forest (CF) has been suggested as an alternative to RF [23].

**Figure 2:**
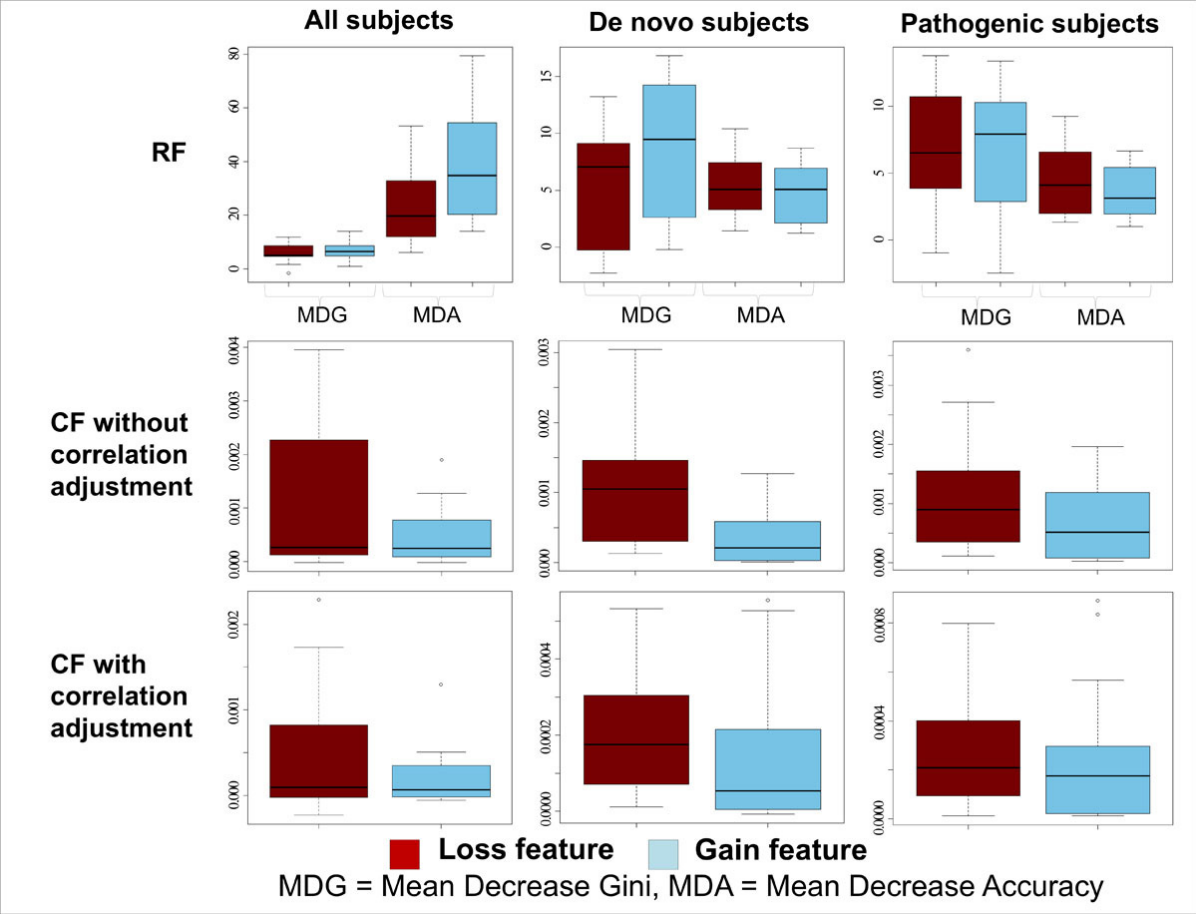
**RF and CF feature relevance, boxplots for the 20 curated neurally-relevant features.** Feature relevance boxplots for loss-based features (red) and gain-based features (blue). Mean decrease gini (MDG) and Mean decrease accuracy (MDA) were used for RF. MDA, with and without correlation adjustment, was used for CF. For all relevance metrics, higher values correspond to more relevant features.

The performance of RF and CF was compared for the 20 curated neurally-relevant features plus the total gene count. We found CF to have slightly higher AUC than RF when classifying all subjects (all CNV types, loss-only, gain-only), whereas it had slightly lower AUC than RF for *de novo* and pathogenic subjects (Table 2). We inspected the feature relevance metrics for both classifiers, using the Mean Decrease Accuracy and Mean Decrease Gini for RF, and Mean Decrease Accuracy, with or without correlation adjustment, for CF. Both RF metric showed greater relevance for gain-based features when classifying all subjects, in contradiction with the fact that classification based uniquely on losses displayed a better AUC than the one based on gains. On the other hand, CF relevance metrics showed the opposite pattern, with loss-based features more relevant than gain-based features, in accordance with expectations (Figure 2). For this reason, we used CF in place of RF for the rest of the analyses.

**Table 2:** CF and RF classification performance for 20 neurally-relevant curated features (mean ± sd)

Subject	Classifier	All CNV	Gain CNV	Loss CNV
All subjects	RandomForest	0.531±0.005	0.509±0.004	0.544±0.006

All subjects	CForest	0.533±0.004	0.513±0.005	0.546±0.003

De novo	RandomForest	0.805±0.012	0.769±0.024	0.840±0.010

De novo	CForest	0.787±0.008	0.732±0.013	0.846±0.011

Pathogenic	RandomForest	0.913±0.014	0.913±0.012	0.935±0.016

Pathogenic	CForest	0.880±0.012	0.897±0.008	0.922±0.030

The AUC achieved by CF using the 20 curated neurally-relevant features and the total count was greater by several standard deviation (sd) units than the AUC achieved using the total gene count alone; it was also greater than the AUC achieved by the total gene count plus 20 matched randomized features obtained by permuting the gene identities and re-computing the gene-set counts for gains and losses. This was particularly the case when classifying all subjects; on the other hand, when classifying only *de novo* or pathogenic CNV carriers versus controls, gains displayed an AUC close to the AUC obtained using the total gene count alone. This can be interpreted in relation to the larger size of pathogenic and *de novo* gains compared to control gains. In addition, as expected, adding the 20 randomized features to the total gene count did not lead to a remarkable increase of the AUC (i.e. lower than or within one sd unit) (Table 3).

**Table 3:** Classification results for all subjects using 20 neurally-relevant curated features, 20 matched randomized features, Gene Ontology and pathways (mean ± sd)

Gene set (All subjects)	All CNV	Gain CNV	Loss CNV
20 curated	0.533±0.004	0.513±0.005	0.546±0.003

GO	0.512±0.005	0.506±0.002	0.519±0.002

GO (man. selected)	0.520±0.005	0.505±0.005	0.524±0.003

GO (f.s.: 20 MDA dec.)	0.524±0.003	0.510±0.003	0.529±0.005

Pathway	0.500±0.000	0.500±0.000	0.504±0.004

Pathway (man. selected)	0.500±0.000	0.500±0.001	0.510±0.004

Pathway (f.s.: 20 MDA dec.)	0.513±0.003	0.510±0.004	0.513±0.003

Random (20 curated)	0.517±0.005	0.510±0.007	0.515±0.007

Total count	0.515±0.005	0.505±0.005	0.516±0.004

CF classification using the 20 curated neurally-relevant features (gains and losses), together with the total gene count, resulted in 7.9% correctly classified ASD subjects and less than 3% incorrectly classified control; this result is reasonably close to the expected contribution of rare CNVs to ASD risk in 10% of the ASD subjects. Losses alone correctly classified 5.9% of the ASD subjects and less than 2.3% incorrectly classified controls; this suggests that gains, besides the ones already recognized to be pathogenic, have limited predictive power, a result in line with univariate burden analysis for this data-set [32].

A detailed analysis of feature relevance for all subjects, or only *de novo* and pathogenic, is presented in the supplementary methods and results [see Additional file 1].

### Classification results for GO and pathways using different feature selection strategies

The set of features based on GO annotations and pathways presented the additional challenges of having many features, with a high degree of mutual overlap and presence of many features with limited or no classification relevance.

To address those issues, we performed classification based on GO and pathway features in two ways: (a) using all features, (b) embedding a feature selection step in the cross-validation procedure; in particular, we used (i) Mean Decrease Accuracy (MDA) based selection, (ii) MDA based selection with stepwise decorrelation, (iii) MRMR (Minimum Redundancy Maximum Relevance Feature Selection). For each procedure, we selected the top 20, top 15% and top 40% ranking features excluding the total gene count, and then added the total gene count. It is important to point out that feature selection was based on the feature relevance metrics calculated on the data subset used for training, and performed independently for every training set, to avoid any overfitting issues.

We assessed the classification performance of different feature selection strategies in comparison to classification without any extra feature selection step or performing manual feature selection based on previous knowledge of ASD biology.

GO without feature selection produced a suboptimal performance (the AUC was very similar to using the gene count alone). When classifying all subjects, the best results for GO were achieved by MDA, either by taking the top 20 features using MDA with decorrelation or by taking the top 15% using MDA without decorrelation; after decorrelation, the top 15% features had a lower performance, suggesting that many relevant yet highly correlated features are removed by decorrelation (see Additional file 1). The best feature selection strategy achieved a slightly better performance than the manually selected GO sub-set (1 sd unit or more), but still inferior to the 20 curated neurally-relevant features (Table 3).

The performance for pathway-based features was markedly worse, with the AUC very close to the total gene count, even when restricting to the manually-selected pathway subset and with the best feature selection strategy (top 20 features using MDA and decorrelation) (Table 3).

These results suggest that feature selection is able to manage the large number and redundancy of GO and pathway-based, although their information content for ASD classification based on rare CNV genes appears to be more limited than the curated neurally-relevant features. Pathway-based features may have particularly disappointing performance results because of the small size of the corresponding gene-sets. Based on these results, we preferred using only the 20 curated neurally-relevant features for the other analyses. It is important to consider that the 20 curated neurally-relevant features include two features derived from the gene-set union of the GO and pathways manually-selected on the basis of known ASD pathobiology.

### Classification results using other classifiers and modifying CF parameters

Classification of all subjects using linear SVM or NN achieved lower or comparable AUC compared to CF [see Additional file 1]. This suggests CF is an optimal classification method for this problem.

Additional analysis demonstrated that CF performance does not vary substantially modifying model construction settings (inferential tests and p-value threshold for feature selection operating for tree construction) [see Additional file 1].

Finally, we compared the performance of our classifier based on the 20 neurally-relevant feature to an existing classifier designed to distinguish benign from pathogenic CNV for mental retardation (GeCCO [19]). We found our classifier to have a significantly better performance [see Additional file 1].

### Prioritization of subjects

We used the classification results based on the 20 curated neurally-relevant features and total gene count to prioritize subjects with potentially interesting inherited rare losses; we focused on inherited rare variation because it is more difficult to assess its significance outside known ASD loci, and we selected rare losses as these display better classification performance than rare gains.

To ensure the classification results are robust to the algorithm’s stochasticity, we required a subject to be classified in at least 15 cross-validation iterations out of 20. We scanned the prediction probability cutoffs to maximize the number of correctly predicted ASD subjects without pathogenic or *de novo* CNVs with respect to incorrectly classified controls. At the ASD prediction probability cutoff of 0.52, the correctly classified ASD subjects represented 3.76% of all ASD subjects with at least one genic rare loss, which dropped to 1.25% (12 subjects) when removing subjects with pathogenic or *de novo* rare CNVs (TP*); this was still larger (about 3.5x) than 0.36% of incorrectly classified controls (FP, false positives), interpretable as a false discovery rate < 30% [see Additional file 1].

We manually inspected these 12 subjects, and found that one of them had a pathogenic loss missed by the clinical annotation, whereas the others had at least one VUS (Variant of Unknown Clinical Significance) each; we used a set of top-scoring loss-based features to identify specific genes that are more likely to contribute to ASD risk within the VUS variant [see Additional file 5].

Finally, we also noticed that prioritized subjects were significantly enriched in females compared to all autism subjects with at least one rare genic loss (Fisher’s Exact Test p-value 0.0066 and OR 2.7). A similar enrichment was reported when considering pathogenic CNVs further classified as “highly penetrant” based on clinical genetic literature [32].

## Conclusions

We successfully used rare CNVs and neurally-relevant gene annotations to classify ASD subjects: the best classifier achieved an AUC of 0.533, corresponding to 7.9% ASD subjects correctly classified by rare CNVs and less than 3% incorrectly classified controls; this result is reasonably close to prior expectations that about 10% of ASD subjects have rare CNV contributing to ASD risk.

Conditional Inference Forest (CF) typically performed as well as or better than other classifiers, and was also found to have a stable performance when using parameter settings different than defaults. Losses alone displayed a markedly stronger classification power than gains; in addition, features based on brain expression, synaptic component and neuro-phenotypes had a superior performance to the full collection of GO and pathways, even after the latter were pre-processed by feature selection and de-correlation methods. This classification approach can be used for other case-control rare CNV data-sets; the features we found to be optimal for ASD are likely to perform well for other neurodevelopmental and neuropsychiatric disorders (e.g. developmental delay, schizophrenia), which also display a specific burden of rare variation for neuronal and neurodevelopmental genes [54-57].

The CF classifier based on neurally-relevant features was also successfully used to extract subjects with inherited losses potentially contributing to ASD risk, but not classified as pathogenic CNVs according to clinical annotation. Nonetheless, since classification performance is particularly high for pathogenic and *de novo* CNV carriers, this type of analysis can also be used to prioritize subjects in the absence of clinical annotations.

Within this classification framework, improved performance could perhaps be achieved by (i) using additional indexes of genic intolerance to variation [58] (ii) modeling the different level of constraint for different gene components (coding exons, introns, UTRs), (iii) expanding the gene annotation feature set, for instance using gene interaction network distances from known ASD disease genes [59], (iv) modeling non-coding RNAs, regulatory sequences and other inter-genic elements [60]), (v) weighting differently X-linked variants based on the subjects’ genders. Modeling gender more accurately is of particular interest, considering the higher prevalence of autism in males compared to females; it would be ideal to train and assess the performance of the classifier for male and female subjects separately, which was not possible for our data-set owing to the small number of female subjects.

## Availability of supporting data

The complete set of stage-1 CNV calls is currently available in dbGAP as phs000267.v3.p2; stage-2 will be soon available in dbGAP as phs000267.v4.p2. Rare variants for ASD subjects and controls are provided as additional files [see Additional file 3-4]

## List of abbreviations used (if any)

ASD: Autism Spectrum Disorders; AUC: Area Under the ROC Curve; CF: Conditional Inference Forest; CNV: Copy Number Variant; GO: Gene Ontology; HPO: Human Phenotype Ontology; MDA: Mean Decrease Accuracy; MGI: Mouse Genome Informatics; MPO: Mammalian Phenotype Ontology; MRMR: Minimum Redundancy Maximum Relevance Feature Selection; NN: Neural Network; OOB: Out Of Bag; RF: Random Forest; SD: Standard Deviation; SVM: Support Vector Machine(s); VUS: Variant of Unknown Clinical Significance

## Competing interests

The authors declare that they have no competing interests.

## Authors' contributions

D.M., J.H.C. and S.W.S. designed the study. D.M. and A.C.L. collected and curated data. W.E. and K.D. performed the computational analysis. D.M., W.E. and K.D. drafted the manuscript. All authors helped revising the manuscript and accepted its final version.

## Supplementary Material

Additional file 1 Supplementary methods and results. Click here for file

Additional file 2 **Twenty curated neurally-relevant feature genes** Gene-set names and genes (entrez gene ID) used to construct the twenty curated neurally-relevant features. Click here for file

Additional file 3 **Rare CNV data for ASD subjects** Rare CNV data for ASD subjects. Click here for file

Additional file 4 **Rare CNV data for controls** Rare CNV data for controls. Click here for file

Additional file 5 **Prioritized ASD subjects carrying rare inherited losses** Subject IDs, CNV coordinates, CNV clinical annotation, CNV gene mapping and gene annotations for the 12 prioritized ASD subjects carrying rare inherited losses potentially contributing to ASD risk. Click here for file

## Declarations

The article publication fee was funded by the Science and Technology Innovation Centre (STIC) technology development funding from Genome Canada/Ontario Genomics Institute.

This article has been published as part of *BMC Medical Genomics* Volume 8 Supplement 1, 2015: Selected articles from the 2nd International Genomic Medical Conference (IGMC 2013): Medical Genomics. The full contents of the supplement are available online at http://www.biomedcentral.com/bmcmedgenomics/supplements/8/S1.b29
