# Supplementary material for: Performance of case-control rare copy number variation annotation in classification of autism
Source: BMC Med Genomics. 2015 Jan 15;8(Suppl 1):S7. doi: 10.1186/1755-8794-8-S1-S7 (PMC4315323; doi:10.1186/1755-8794-8-S1-S7)
Supplement: Additional file 1 — Supplementary methods and results. [file 1755-8794-8-S1-S7-S1.pdf]

SUPPLEMENTARY METHODS AND RESULTS

Supplementary methods

Gene-sets used for feature construction

hi015: genes with predicted haploinsufficiency score [R1] >= 0.15 (most sensitive cutoff)
hi035: genes with predicted haploinsufficiency score [R1] >= 0.35 (best tradeoff between sensitivity and specificity)
hi055: genes with predicted haploinsufficiency score [R1] >= 0.55 (most specific cutoff)
ExpsNov_BrainFeAd_sp: genes specifically expressed in fetal or adult brain, defined as: rma expression index for the fetal or adult brain greater than the median expression for the entire data-set and greater than twice the median expression of non-brain tissue; based on the Novartis Tissue Expression Atlas (U133A Affymetrix array) [R2]
Synapse_GrantFull: full list of post-synaptic density components based on human neocortex proteomics [R3]
FMR1_Targets_Darnell: human orthologs (NCBI Homologene) of mouse genes whose mRNA translation in neurons is likely to be regulated by the FMR1 protein, based on crosslinking immunoprecipitation (HITS-CLIP) of mouse brain polyribosomal mRNAs [R4]
FMR1_Targets_Ascano: genes whose mRNA translation in neurons is likely to be regulated by the FMR1 protein, based on bioinformatics prediction supported by regulatory sequence motifs [R5]
thr4.86_log2rpkm: genes with at least 5 BrainSpan [R6] data points for which log2 (rpkm) >= 4.86, thus deemed expressed at (very) high levels in brain.
thr3.32_log2rpkm: genes with at least 5 BrainSpan [R6] data points for which 4.86 > log2 (rpkm) >= 3.32, thus deemed expressed at high/medium levels in brain.
thr0.84_log2rpkm: genes with at least 5 BrainSpan [R6] data points for which 3.32 > log2 (rpkm) >= 0.84, thus deemed expressed at medium/low levels in brain.
thr.MIN_log2rpkm: genes with BrainSpan [R6] data points failing all previous criteria, thus deemed expressed at very low level or not expressed in brain.
thrEXPR_log2rpkm: union of genes in the sets thr4.86_log2rpkm, thr3.32_log2rpkm, thr0.84_log2rpkm, thus deemed expressed in brain
PhHs_NervSys_ADX: genes implicated in human disorders with abnormality of the nervous system, autosomal dominant or X-linked mode of inheritance, downloaded from HPO (Human Phenotype Ontology) [R7] in June 2013.
PhHs_NervSys_All: genes implicated in human disorders with abnormality of the nervous system, any mode of inheritance, downloaded from HPO (Human Phenotype Ontology) [R7] in June 2013.
PhHs_MindFun_ADX: genes implicated in human disorders with abnormality of higher mental function, autosomal dominant or X-linked mode of inheritance, downloaded from HPO (Human Phenotype Ontology) [R7] in June 2013.
PhHs_MindFun_All: genes implicated in human disorders with abnormality of higher mental function, any mode of inheritance, downloaded from HPO (Human Phenotype Ontology) [R7] in June 2013.

MmHs_Neuro_All: genes whose knock out (or other genetic construct) produces a (a) nervous system or (b) behavior/neurological phenotype in mouse, downloaded from MGI (Mouse Genome Informatics) [R8] in June 2013.
MmHs_Extend_All: genes whose knock out (or other genetic construct) produces (a) embryogenesis or (b) growth/size/body or (c) craniofacial phenotype in mouse, downloaded from MGI (Mouse Genome Informatics) [R8] in June 2013.
NeuroF_large: genes in at least one of the curated Gene Ontology and pathway derived sets of neurobiological relevance
NeuroF_small: genes in at least two of the curated Gene Ontology and pathway derived sets of neurobiological relevance

The following list of Gene Ontology and pathway-derived sets of neurobiological relevance was used for the definition of NeuroF_large and NeuroF_small, as well as for the assessment of GO and pathway feature selection: GO:0007399 nervous system development, GO:0019227 neuronal action potential propagation, GO:0019226 transmission of nerve impulse, GO:0050890 cognition, GO:0045202 synapse, GO:0043005 neuron projection, GO:0043025 neuronal cell body, Reactome Neuronal System, Reactome NCAM signaling for neurite out-growth, Reactome Axon guidance, KEGG:04725 Cholinergic synapse, KEGG:04724 Glutamatergic synapse, KEGG:04728 Dopaminergic synapse, KEGG:04727 GABAergic synapse, KEGG:04726 Serotonergic synapse, KEGG:04721 Synaptic vesicle cycle, KEGG:04723 Retrograde endocannabinoid signaling, KEGG:04720 Long-term potentiation, KEGG:04730 Long-term depression, KEGG:04722 Neurotrophin signaling pathway, KEGG:04360 Axon guidance, NCI Netrin Pathway, NCI Reelin Pathway (downloaded from Gene Ontology, KEGG, Reactome and NCI June 2013).

Feature selection with stepwise decorrelation for GO and pathway features (CF)

1. Given the set of all features F = {f1, f2, …, fn}, where n is the total number of features
2. Calculate the Mean Decrease Accuracy for each feature and rank features in decreasing order
3. Select the feature with top rank
4. Binarize features (by setting gene count values greater than 1 to 1), and calculate pairwise Jaccard similarity as J (fi, fj) = (sum (fi AND fj) / sum (fi OR fj)), where AND and OR are the element-wise logical operators, and logical values are expressed as TRUE = 1 and FALSE = 0 (this formulation is equivalent to the set operator based definition, but perhaps more intuitive for binary vectors)
5. Remove all features that have lower ranks than the selected feature and similarity to the selected feature above the cutoff of 0.5
6. Repeat step 3-5 until reaching the desired number of selected features, or until no feature is left (step 4 does not need to be repeated, as Jaccard similarities do not change)

MRMR Feature selection for GO and pathway features (CF)

MRMR (Minimum Redundancy Maximum Relevance Feature Selection): features were ranked based on the MRMR method, an effective approach for large feature sets with high degree of mutual redundancy and noisiness, where only a small unknown subset of features are truly discriminative. Features are typically selected one at a time by finding the next feature from the unselected set displaying minimal redundancy with the set of features already selected and maximal relevance to the true class labels; the first selected feature is maximally relevant to the class labels. In this study, we scored each feature fi using the ratio between D = I (FS, Y) and R = I (FS, fi), where I represents the mutual information function, Y represents the subject's class (ASD = 1, control = 0), FS represents the set of selected features; therefore, D represents the relevance of the feature being evaluated with respect to the true class labels, whereas R represents the redundancy of the feature being evaluated with respect to the features already selected. It can be proven mathematically that the resulting feature set is maximally dependent on the true class labels and has a reduced correlational structure compared with the original feature set.

Feature selection for GO and pathway features (Linear SVM, Neural Network)

Features were selected using only the MRMR D / I ratio described above. For each cross-validation iteration, the number of selected features was chosen at performance saturation (i.e. selecting more features leads to a similar or lower performance).

<u>Supplementary results</u>

Feature relevance for 20 curated features capturing brain expression, synaptic components, neuro-phenotypes

Carefully inspecting the CF feature relevance metrics, and specifically comparing the results when classifying all case subjects to de-novo or pathogenic case only, we identified several meaningful patterns:

1. feature relevance is overall similar when classifying all subjects or only de novo or pathogenic CNV carriers, although with notable excpetions
2. features based on medium size sets (750-5,000 genes), such as synaptic components (FMR1_Targets_Darnell, Synapse_GrantFull), high brain expression (thr4.86_log2rpkm), and mouse neuro-phenotypes (MmHs_Neuro_All), typically have higher relevance score for all subjects than for de-novo or pathogenic case subjects only;
3. features based on larger sets (> 5,000 genes), such as all brain expressed (thrEXPR_log2rpkm), moderate to high predicted haploinsufficiency (hi015), total count (Total), have higher for relevance score for de-novo and pathogenic case subjects than all subjects, especially for losses; this can be interpreted in relation to the larger size (and number of genes) of de novo and pathogenic CNVs;
4. haploinsufficiency features are more relevant for losses than for gains, which is expected based on the definition of haploinsufficiency as sensitivity to decreased gene product dosage;
5. features based on smaller sets (< 700-800 genes), such as human neurological

phenotype genes (PhHs_...), are less relevant, probably because they account for a smaller number of subjects, and most of their genes are already present in other better ranked gene-sets;

6. the feature based on very low or absent brain expression set (thr.MIN_log2rpkm) ranks in the bottom half for both gains and losses

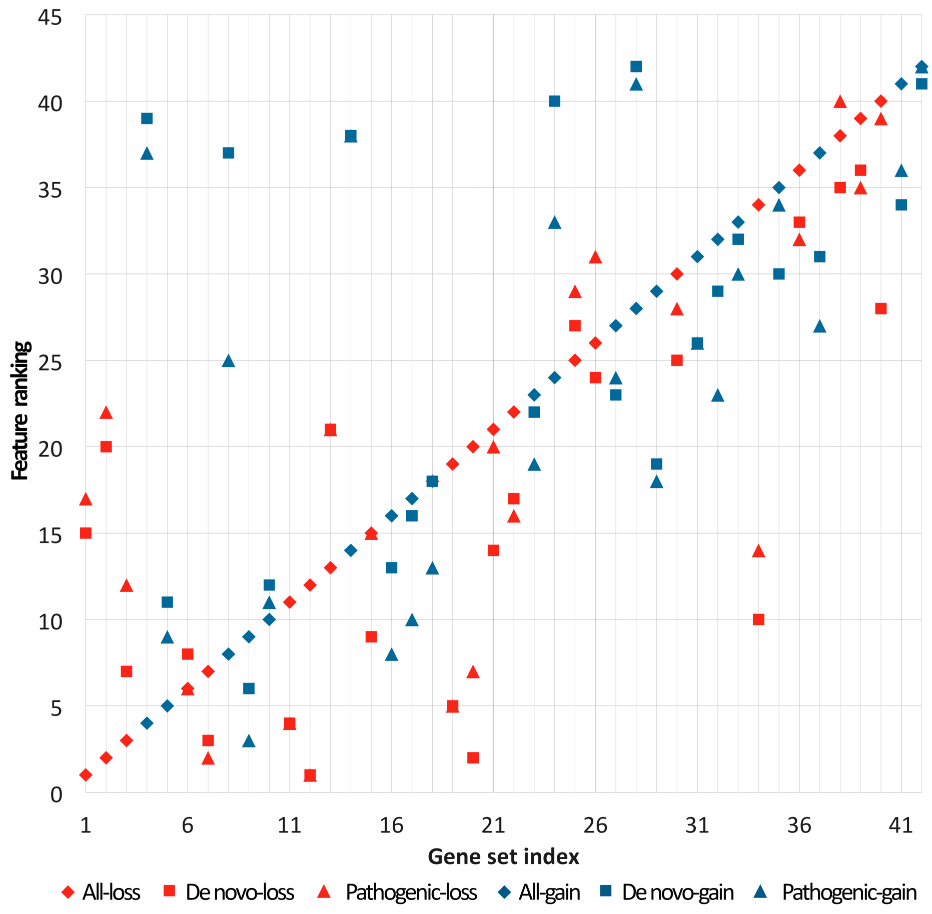

ID	Feature name
1	thr4.86_log2rpkm_loss
2	ExprNov_BrainFeAd_sp_loss
3	Synapse_GrantFull_loss
4	FMR1_Targets_Ascano_gain
5	thrEXPR_log2rpkm_gain
6	MmHs_Neuro_All_loss
7	thrEXPR_log2rpkm_loss
8	FMR1_Targets_Darnell_gain
9	thr4.86_log2rpkm_gain
10	MmHs_Extend_All_gain
11	hi035_loss
12	hi015_loss
13	FMR1_Targets_Ascano_loss
14	thr3.32_log2rpkm_gain
15	FMR1_Targets_Darnell_loss
16	MmHs_Neuro_All_gain
17	Total_gain
18	hi015_gain
19	Total_loss
20	MmHs_Extend_All_loss
21	thr3.32_log2rpkm_loss
22	NeuroF_large_loss
23	NeuroF_large_gain
24	Synapse_GrantFull_gain
25	thr.MIN_log2rpkm_loss
26	thr0.84_log2rpkm_loss
27	PhHs_NervSys_ADX_gain
28	ExprNov_BrainFeAd_sp_gain
29	NeuroF_small_gain
30	NeuroF_small_loss
31	thr0.84_log2rpkm_gain
32	hi055_gain
33	hi035_gain
34	hi055_loss
35	PhHs_NervSys_All_gain
36	PhHs_NervSys_All_loss
37	PhHs_MindFun_ADX_gain
38	PhHs_MindFun_ADX_loss
39	PhHs_NervSys_ADX_loss
40	PhHs_MindFun_All_loss
41	PhHs_MindFun_All_gain
42	thr.MIN_log2rpkm_gain

GO and pathway feature selection results

We first assessed performance using the size-filtered Gene Ontology and pathway collection, without any extra feature selection step. We assessed performance in comparison to a manually selected subset of Gene Ontology sets and pathways of neurobiological relevance, thus more likely to contribute to ASD risk; we always included the total gene count as a feature.

We found that using all size-filtered Gene Ontology sets leads to a suboptimal performance: the AUC was slightly lower or within one sd unit of the AUC using the total gene count only, and also slightly lower than using the manually-selected Gene Ontology subset; in contrast, the 20 curated neurally-relevant features and total gene count achieved an AUC that is larger by several sd units. The performance for pathways was markedly worse, with the AUC very close

to 0.5 even when classifying de-novo or pathogenic carriers only; restricting to the manually-selected pathway subset led only to minor improvements. This trend was consistent for the different sub-groups of cases (all subjects vs de-novo or pathogenic variant carriers, all variants vs loss-only or gain-only).

Three feature selection procedures were adopted and compared: (i) feature relevance (Mean Decrease Accuracy, MDA) based selection, (ii) feature relevance (Mean Decrease Accuracy) based selection with stepwise decorrelation, (c) MRMR (Minimum Redundancy Maximum Relevance Feature Selection). For each procedure, we selected the top 20, top 15% and top 40% ranking features excluding the total gene count, and then added the total gene count.

When classifying all subjects, the best results for Gene Ontology based features were achieved by Mean Decrease Accuracy, either by taking the top 15% without decorrelation, or taking the top 20 features with decorrelation. After decorrelation, the top 15% had a lower performance, suggesting that many relevant yet highly correlated features are removed by decorrelation. The best feature selection strategy achieved a slightly better performance than the manually-selected Gene Ontology subset (1 sd unit or more), but still inferior to the 20 curated neurally-relevant features.

When classifying all subjects, the best results for pathway based features were achieved by Mean Decrease Accuracy, taking the top 20 features, with performance quite independent of decorrelation; this is reasonable, considering that pathway derived gene-sets have less mutual overlap than Gene Ontology derived gene-sets, and this is reflected on feature correlation. Also for pathways, the best feature selection strategy achieved a slightly better performance than the manually-selected pathway subset, yet very modest, suggesting that pathways have a limited classification power.

Table S1. Classification results for de novo CNV subjects using 20 neurally-relevant curated features, randomized features, Gene Ontology and pathways.

Gene set (De novo)	All CNV	Gain CNV	Loss CNV
20 curated	0.787±0.006	0.720±0.010	0.847±0.001
GO	0.617±0.011	0.694±0.016	0.733±0.010
GO (man. sel.)	0.714±0.016	0.697±0.010	0.745±0.017
Pathway	0.534±0.033	0.568±0.037	0.500±0.000
Pathway (man. sel.)	0.538±0.018	0.618±0.042	0.510±0.005
Random (20 curated)	0.732±0.016	0.727±0.018	0.708±0.017
Total count	0.744±0.013	0.720±0.016	0.712±0.012

Table S2. Classification results for pathogenic CNV subjects using 20 neurally-relevant curated features, randomized features, Gene Ontology and pathways.

Gene set (Pathogenic)	All CNV	Gain CNV	Loss CNV
20 curated	0.879±0.013	0.887±0.007	0.911±0.029
GO	0.848±0.019	0.817±0.037	0.873±0.026
GO (man. sel.)	0.844±0.010	0.921±0.011	0.857±0.012
Pathway	0.500±0.000	0.510±0.012	0.500±0.000
Pathway (man. sel.)	0.500±0.000	0.602±0.031	0.500±0.000
Random (20 curated)	0.816±0.011	0.866±0.012	0.813±0.013

Total count	0.833±0.011	0.874±0.010	0.802±0.014

Table S3. Results for different feature selection strategies (GO)

Subject	CNV	# features	MDA, without Decorrelation	MDA, with Decorrelation	MRMR
All subjects	All CNV	20	0.512±0.005	0.524±0.003	0.517±0.003
		15%	0.523±0.003	0.520±0.003	0.506±0.005
		40%	0.520±0.002	0.516±0.006	0.503±0.004
	Gain CNV	20	0.510±0.003	0.510±0.003	0.509±0.004
		15%	0.508±0.005	0.509±0.005	0.506±0.003
		40%	0.507±0.003	0.507±0.002	0.503±0.002
	Loss CNV	20	0.529±0.004	0.529±0.005	0.520±0.005
		15%	0.529±0.004	0.520±0.002	0.514±0.002
		40%	0.521±0.002	0.518±0.002	0.515±0.002
De novo	All CNV	20	0.660±0.029	0.654±0.033	0.641±0.031
		15%	0.624±0.007	0.624±0.012	0.650±0.024
		40%	0.632±0.007	0.617±0.009	0.632±0.018
	Gain CNV	20	0.700±0.016	0.700±0.017	0.689±0.024
		15%	0.700±0.018	0.700±0.014	0.701±0.017
		40%	0.691±0.019	0.697±0.014	0.701±0.013
	Loss CNV	20	0.743±0.029	0.758±0.018	0.737±0.023
		15%	0.732±0.01	0.725±0.014	0.752±0.013
		40%	0.733±0.012	0.731±0.016	0.746±0.014
Pathogenic	All CNV	20	0.768±0.034	0.789±0.040	0.811±0.047
		15%	0.799±0.04	0.786±0.051	0.821±0.028
		40%	0.697±0.046	0.847±0.024	0.829±0.016
	Gain CNV	20	0.860±0.036	0.859±0.041	0.854±0.037
		15%	0.812±0.039	0.822±0.021	0.853±0.029
		40%	0.817±0.038	0.804±0.028	0.848±0.043
	Loss CNV	20	0.866±0.021	0.868±0.018	0.877±0.020
		15%	0.870±0.028	0.861±0.035	0.872±0.024
		40%	0.862±0.042	0.876±0.037	0.891±0.016

Table S4. Results for different feature selection strategies (Pathways)

Subject	CNV	# features	MDA, without Decorrelation	MDA, with Decorrelation	MRMR
All subjects	All CNV	20	0.512±0.003	0.513±0.003	0.501±0.002
		15%	0.501±0.002	0.508±0.004	0.500±0.000
		40%	0.500±0.000	0.500±0.000	0.500±0.000
	Gain CNV	20	0.510±0.002	0.510±0.004	0.502±0.002
		15%	0.507±0.005	0.504±0.002	0.500±0.000
		40%	0.500±0.001	0.500±0.000	0.500±0.000
	Loss CNV	20	0.514±0.002	0.513±0.003	0.511±0.005
		15%	0.510±0.002	0.514±0.005	0.508±0.005
		40%	0.507±0.004	0.507±0.004	0.508±0.004
De novo	All CNV	20	0.599±0.030	0.601±0.026	0.616±0.029
		15%	0.520±0.029	0.562±0.036	0.609±0.042
		40%	0.503±0.010	0.568±0.025	0.603±0.043
	Gain CNV	20	0.588±0.044	0.590±0.037	0.658±0.042

		15%	0.581±0.034	0.586±0.028	0.671±0.039
		40%	0.550±0.032	0.581±0.049	0.629±0.054
	Loss CNV	20	0.641±0.040	0.628±0.036	0.625±0.040
		15%	0.532±0.027	0.532±0.033	0.515±0.024
		40%	0.500±0.000	0.504±0.013	0.507±0.017
Pathogenic	All CNV	20	0.617±0.052	0.617±0.052	0.559±0.060
		15%	0.500±0.000	0.507±0.026	0.506±0.021
		40%	0.500±0.000	0.500±0.000	0.500±0.000
	Gain CNV	20	0.690±0.081	0.699±0.081	0.688±0.043
		15%	0.577±0.024	0.584±0.034	0.624±0.042
		40%	0.508±0.013	0.550±0.022	0.565±0.029
	Loss CNV	20	0.661±0.040	0.661±0.040	0.629±0.033
		15%	0.515±0.020	0.515±0.020	0.514±0.016
		40%	0.500±0.000	0.500±0.000	0.500±0.002

Results with other classifiers

Using the same cross-validation strategy as for RF and CF, and the 20 curated neurally-relevant features, both the linear SVM and NN achieved comparable or lower AUC than CF. We did not find evidence of overfitting, as the AUC obtained using the randomization of the 20 curated neurally-relevant features was similar to the AUC of the total gene count.
For both the neural network and the linear SVM, GO and pathway-based features produced better performance than using CF, yet still inferior to the 20 curated features. However, we found potential evidence of some modest degree of overfitting for GO and pathway-based features, as the AUC for random features exceeded by more than one AUC absolute unit (and more than two AUC standard deviation units) the AUC for the total gene count only, and was also very close to the AUC of real Gene Ontology features.

Table S5. Linear SVM Results

Subject	Gene set	All CNV	Gain CNV	Loss CNV
All subjects	20 curated	0.530±0.003	0.509±0.003	0.541±0.004
	GO (MRMR)	0.527±0.007	0.521±0.006	0.527±0.005
	Pathway (MRMR)	0.529±0.004	0.522±0.004	0.529±0.007
	Total Count	0.501±0.001	0.502±0.001	0.510±0.001
	Random (20 curated)	0.514±0.004	0.507±0.002	0.515±0.007
	Random (GO)	0.522±0.007	0.515±0.008	0.526±0.009
	Random (Pathway)	0.522±0.006	0.511±0.006	0.521±0.009
De novo	20 curated	0.647±0.011	0.650±0.018	0.655±0.008
	GO (MRMR)	0.647±0.016	0.654±0.027	0.672±0.020
	Pathway (MRMR)	0.629±0.012	0.654±0.021	0.655±0.020
	Total Count	0.500±0.000	0.500±0.000	0.576±0.019
	Random (20 curated)	0.584±0.018	0.582±0.044	0.601±0.017
	Random (GO)	0.624±0.017	0.626±0.023	0.634±0.023

	Random (Pathway)	0.612±0.018	0.629±0.033	0.622±0.024
Pathogenic	20 curated	0.771±0.019	0.839±0.033	0.747±0.017
	GO (MRMR)	0.797±0.017	0.852±0.048	0.817±0.024
	Pathway (MRMR)	0.760±0.023	0.860±0.027	0.733±0.028
	Total Count	0.500±0.000	0.576±0.024	0.651±0.033
	Random (20 curated)	0.706±0.027	0.800±0.027	0.697±0.030
	Random (GO)	0.764±0.028	0.866±0.046	0.754±0.037
	Random (Pathway)	0.753±0.026	0.845±0.042	0.731±0.040

Table S6. Neural Network Results

Subject	Gene set	All CNV	Gain CNV	Loss CNV
All subjects	20 curated	0.516±0.005	0.513±0.009	0.531±0.007
	GO (MRMR)	0.527±0.007	0.519±0.009	0.529±0.006
	Pathway (MRMR)	0.527±0.007	0.520±0.009	0.530±0.005
	Total Count	0.503±0.004	0.504±0.006	0.514±0.007
	Random (20 curated)	0.514±0.006	0.509±0.010	0.518±0.008
	Random (GO)	0.516±0.009	0.513±0.007	0.525±0.005
	Random (Pathway)	0.516±0.007	0.509±0.007	0.522±0.005
De novo	20 curated	0.653±0.015	0.631±0.035	0.691±0.020
	GO (MRMR)	0.641±0.009	0.638±0.032	0.665±0.025
	Pathway (MRMR)	0.637±0.019	0.641±0.032	0.662±0.018
	Total Count	0.528±0.018	0.581±0.043	0.638±0.013
	Random (20 curated)	0.620±0.036	0.607±0.037	0.620±0.028
	Random (GO)	0.625±0.025	0.638±0.028	0.637±0.022
	Random (Pathway)	0.621±0.025	0.639±0.039	0.636±0.012
Pathogenic	20 curated	0.774±0.024	0.815±0.045	0.822±0.037
	GO (MRMR)	0.760±0.021	0.839±0.041	0.806±0.048
	Pathway (MRMR)	0.752±0.028	0.862±0.046	0.790±0.046
	Total Count	0.554±0.020	0.710±0.040	0.709±0.057
	Random (20 curated)	0.689±0.027	0.782±0.046	0.698±0.057
	Random (GO)	0.736±0.032	0.840±0.055	0.747±0.043
	Random (Pathway)	0.728±0.031	0.847±0.043	0.721±0.042

CF robustness to parameter change

For the 20 curated neurally-relevant features, we tested different inferential statistics used by CF for tree construction and observed minor differences in performance; the default settings (Teststat = max, Testtype = Teststatistic) usually had the best performance, or performance

comparable to other settings (i.e. within one sd unit). Similarly, we observed minor differences by modifying the “mincriterion” for the default inferential test statistic (default value: 0.9), which corresponds to (1 - test p-value) and needs to be satisfied by all features used for tree construction.

Table S7. Assessment of Testtype/Teststat/mincriterion parameters for 20 curated feature set.

Subject	Parameters	All CNV	Gain CNV	Loss CNV
All subjects	Teststatistic/Quadratic/0.95	0.533±0.005	0.512±0.004	0.548±0.006
	Teststatistic/Max/0.95	0.537±0.004	0.515±0.003	0.548±0.005
	Univariate/Quadratic/0.95	0.530±0.005	0.508±0.003	0.542±0.005
	Univariate/Max/0.95	0.530±0.005	0.508±0.003	0.542±0.005
	Teststatistic/Quadratic/0.9	0.537±0.004	0.513±0.004	0.548±0.005
De novo	Teststatistic/Quadratic/0.95	0.787±0.006	0.720±0.010	0.847±0.011
	Teststatistic/Max/0.95	0.791±0.005	0.724±0.009	0.848±0.009
	Univariate/Quadratic/0.95	0.784±0.020	0.713±0.010	0.838±0.019
	Univariate/Max/0.95	0.784±0.020	0.713±0.010	0.838±0.019
	Teststatistic/Quadratic/0.9	0.786±0.008	0.722±0.011	0.848±0.010
Pathogenic	Teststatistic/Quadratic/0.95	0.879±0.013	0.887±0.007	0.911±0.029
	Teststatistic/Max/0.95	0.88±0.0120	0.887±0.011	0.919±0.027
	Univariate/Quadratic/0.95	0.863±0.018	0.889±0.006	0.908±0.026
	Univariate/Max/0.95	0.863±0.018	0.889±0.006	0.908±0.026
	Teststatistic/Quadratic/0.9	0.875±0.012	0.890±0.007	0.916±0.022

Table S8. Assessment of mincriterion

Subject	mincriterion	All CNV	Gain CNV	Loss CNV
All subjects	0.95	0.533±0.005	0.512±0.004	0.548±0.006
	0.75	0.539±0.004	0.513±0.006	0.552±0.005
	0.5	0.539±0.005	0.514±0.007	0.553±0.006
De novo	0.95	0.787±0.006	0.720±0.010	0.847±0.011
	0.75	0.789±0.009	0.722±0.011	0.844±0.011
	0.5	0.792±0.006	0.725±0.011	0.845±0.011
Pathogenic	0.95	0.879±0.013	0.887±0.007	0.911±0.029
	0.75	0.875±0.018	0.887±0.005	0.908±0.025
	0.5	0.875±0.020	0.888±0.007	0.912±0.026

Classification probability cutoff and TP, FP fractions (used to prioritize 12 subjects with inherited losses potentially contributing to ASD risk)

Table S9. Classification probability cutoff space, with true positive fraction (TP), true positive fraction without pathogenic or de novo carriers (TP*), false positive fraction (FP); fractions are calculated using only subjects with a genic loss

**Freq.**	**>=5**			**>=10**			**>=15**		
**Cutoff**	**TP**	**TP***	**FP**	**TP**	**TP***	**FP**	**TP**	**TP***	**FP**
0.4	1	0.910	1	1	0.910	1	1	0.910	1
0.42	1	0.910	1	1	0.910	1	1	0.910	1

0.44	0.976	0.887	0.972	0.795	0.714	0.738	0.521	0.452	0.437
0.46	0.235	0.182	0.158	0.189	0.138	0.111	0.151	0.102	0.067
0.48	0.123	0.075	0.054	0.092	0.050	0.032	0.077	0.040	0.021
0.5	0.088	0.047	0.028	0.067	0.031	0.017	0.043	0.016	0.013
0.52	0.071	0.034	0.020	0.046	0.019	0.010	**0.038**	**0.013**	**0.004**
0.54	0.054	0.023	0.011	0.040	0.015	0.004	0.032	0.009	0.003
0.56	0.045	0.018	0.007	0.036	0.012	0.003	0.029	0.008	0.002
0.58	0.040	0.014	0.003	0.030	0.009	0.002	0.026	0.006	0.002
0.6	0.034	0.012	0.003	0.028	0.007	0.002	0.025	0.005	0.002
0.62	0.030	0.009	0.002	0.027	0.007	0.002	0.024	0.005	0.001
0.64	0.027	0.007	0.002	0.024	0.005	0.001	0.023	0.005	0.001
0.66	0.026	0.006	0.002	0.023	0.005	0.001	0.023	0.005	0.001
0.68	0.025	0.005	0.002	0.023	0.005	0.001	0.022	0.004	0
0.7	0.023	0.005	0.001	0.023	0.005	0.001	0.020	0.003	0
0.72	0.023	0.005	0.001	0.021	0.003	0.001	0.019	0.003	0
0.74	0.022	0.004	0.001	0.021	0.003	0	0.017	0.001	0
0.76	0.021	0.003	0	0.019	0.003	0	0.015	0.001	0
0.78	0.021	0.003	0	0.018	0.003	0	0.008	0	0
0.8	0.020	0.003	0	0.014	0.001	0	0.006	0	0
0.82	0.017	0.002	0	0.009	0.001	0	0.001	0	0
0.84	0.013	0.001	0	0.002	0	0	0	0	0
0.86	0.002	0	0	0	0	0	0	0	0
0.88	0	0	0	0	0	0	0	0	0
0.9	0	0	0	0	0	0	0	0	0
0.92	0	0	0	0	0	0	0	0	0

References

[R1] Characterising and predicting haploinsufficiency in the human genome.
Huang N, Lee I, Marcotte EM, Hurles ME.
PLoS Genet. 2010 Oct 14;6(10):e1001154.

[R2] A gene atlas of the mouse and human protein-encoding transcriptomes.
Su AI, Wiltshire T, Batalov S, Lapp H, Ching KA, Block D, Zhang J, Soden R, Hayakawa M, Kreiman G, Cooke MP, Walker JR, Hogenesch JB.
Proc Natl Acad Sci U S A. 2004 Apr 20;101(16):6062-7.

[R3] Characterization of the proteome, diseases and evolution of the human postsynaptic density.
Bayés A, van de Lagemaat LN, Collins MO, Croning MD, Whittle IR, Choudhary JS, Grant SG.
Nat Neurosci. 2011 Jan;14(1):19-21.

[R4] FMRP stalls ribosomal translocation on mRNAs linked to synaptic function and autism.
Darnell JC, Van Driesche SJ, Zhang C, Hung KY, Mele A, Fraser CE, Stone EF, Chen C, Fak JJ, Chi SW, Licatalosi DD, Richter JD, Darnell RB.

Cell. 2011 Jul 22;146(2):247-61.

[R5] FMRP targets distinct mRNA sequence elements to regulate protein expression.
Ascano M Jr, Mukherjee N, Bandaru P, Miller JB, Nusbaum JD, Corcoran DL, Langlois C, Munschauer M, Dewell S, Hafner M, Williams Z, Ohler U, Tuschl T.
Nature. 2012 Dec 20;492(7429):382-6.

[R6] BrainSpan: Atlas of the Developing Human Brain [Internet]. Funded by ARRA Awards 1RC2MH089921-01, 1RC2MH090047-01, and 1RC2MH089929-01. © 2011. Available from: http://developinghumanbrain.org.

[R7] Sebastian Köhler,Sandra C Doelken,Christopher J. Mungall,Sebastian Bauer,Helen V. Firth,Isabelle Bailleul-Forestier,Graeme C. M. Black,Danielle L. Brown,Michael Brudno,Jennifer Campbell,David R. FitzPatrick,Janan T. Eppig,Andrew P. Jackson,Kathleen Freson,Marta Girdea,Ingo Helbig,Jane A. Hurst,Johanna Jähn,Laird G. Jackson,Anne M. Kelly,David H. Ledbetter,Sahar Mansour,Christa L. Martin,Celia Moss,Andrew Mumford,Willem H. Ouwehand,Soo-Mi Park,Erin Rooney Riggs,Richard H. Scott,Sanjay Sisodiya,Steven Van Vooren,Ronald J. Wapner,Andrew O. M. Wilkie,Caroline F. Wright,Anneke T. Vulto-van Silfhout,Nicole de Leeuw,Bert B. A. de Vries,Nicole L. Washingthon,Cynthia L. Smith,Monte Westerfield,Paul Schofield,Barbara J. Ruef,Georgios V. Gkoutos,Melissa Haendel,Damian Smedley,Suzanna E. Lewis,and Peter N. Robinson
The Human Phenotype Ontology project: linking molecular biology and disease through phenotype data
Nucl. Acids Res. (1 January 2014) 42 (D1): D966-D974

[R8] www.informatics.jax.org/
